# Palaeoproteomics guidelines to identify proteinaceous binders in artworks following the study of a 15th-century painting by Sandro Botticelli’s workshop

**DOI:** 10.1038/s41598-022-14109-w

**Published:** 2022-06-23

**Authors:** F. Di Gianvincenzo, D. Peggie, M. Mackie, C. Granzotto, C. Higgitt, E. Cappellini

**Affiliations:** 1 Globe Institute, University of Copenhagen, Copenhagen, Denmark; 2 National Gallery Scientific Department, London, UK; 3 Novo Nordisk Foundation Center for Protein Research, University of Copenhagen, Copenhagen, Denmark; 4 Department of Conservation and Science, Art Institute of Chicago, Chicago, IL USA

**Keywords:** Proteomics, Mass spectrometry

## Abstract

Undertaking the conservation of artworks informed by the results of molecular analyses has gained growing importance over the last decades, and today it can take advantage of state-of-the-art analytical techniques, such as mass spectrometry-based proteomics. Protein-based binders are among the most common organic materials used in artworks, having been used in their production for centuries. However, the applications of proteomics to these materials are still limited. In this work, a palaeoproteomic workflow was successfully tested on paint reconstructions, and subsequently applied to micro-samples from a 15th-century panel painting, attributed to the workshop of Sandro Botticelli. This method allowed the confident identification of the protein-based binders and their biological origin, as well as the discrimination of the binder used in the ground and paint layers of the painting. These results show that the approach is accurate, highly sensitive, and broadly applicable in the cultural heritage field, due to the limited amount of starting material required. Accordingly, a set of guidelines are suggested, covering the main steps of the data analysis and interpretation of protein sequencing results, optimised for artworks.

## Introduction

Detailed knowledge of the materials present in an artwork is necessary to reconstruct its history, understand the artists’ techniques, define the best display and storage conditions, and plan appropriate restoration treatments. The exact composition of artistic objects is rarely known a priori, and can potentially include a wide variety of inorganic and organic compounds^1,2^. Contemporaneous written accounts are a useful guide to understanding the materials and techniques used in paintings^2^, however, it is only with the application of instrumental analysis that information on the specific practices of individual artists can be elucidated, and compared with information in written sources from the artist(s) themselves^3^. Moreover, degradation of the materials greatly increases the complexity of the paint matrix, resulting in significant changes to its chemical and physical properties, sometimes affecting the object’s appearance^4^.

Given the cultural and historical importance of artworks, minimally invasive analyses are preferred. Therefore, the first investigational approach is commonly non-invasive, i.e. not requiring any contact with the object^5^. Using non-invasive techniques in the identification of inorganic components is well-established and the results are generally conclusive^6–10^. On the other hand, the results on organic components are less specific, as these techniques can, at best, only allow for the identification of the class(es) of molecules present, e.g. protein, oil, etc., and rarely for the determination of the specific material, e.g. animal glue versus egg^11–15^. Detailed and accurate information about organic materials is generally provided by invasive or destructive techniques, which involve the collection of a sample to be preserved or destroyed after the analysis, respectively^11,16^. Sampling strategies are designed to have as little impact as possible, by minimising the amount of material collected, and by removing micro-samples from areas of existing damage or from the edges of the artwork.

In paintings, protein-based materials can be used as binders in preparation and paint layers, varnishes, protective layers, or adhesives^2,17^, and multiple analytical techniques are used for their detection and analysis^18^. The use of mass spectrometry (MS)-based proteomics approaches for the study of ancient protein residues, i.e. palaeoproteomics, has acquired increasing importance in the scientific community during the last two decades^19^. The first application of MS-based proteomics to ancient materials dates back to 2000^20^, and applications to artworks emerged only a few years later^21,22^. Proteomic approaches allow for the confident identification of ancient protein residues, and for the discrimination of the taxonomical species and tissue of origin^23^, even when a mixture of proteinaceous materials is present. The proteomic characterisation of paint samples from artworks is particularly challenging because: (1) samples are ordinarily collected in sub-milligram amounts, (2) they are complex matrices rich in inorganic materials, such as pigments, with cations known to negatively affect protein recovery^24,25^, (3) the proteinaceous material represents no more than 10% of the paint^26^, and (4) the proteins are often heavily damaged due to age-related degradation. These challenges are illustrated by the fact that, so far, confident identification of proteins and their biological source has been achieved only with samples significantly larger than those routinely used for chromatographic and spectroscopic analyses (up to a few hundreds of micrograms)^22,27–29^.

In this study, a palaeoproteomic workflow developed for archaeological samples^30^ and used previously for unpigmented surface layers of a *fresco* wall painting^31^, and state-of-the-art MS instrumentation were used to assess whether confident protein characterisation could be achieved from small paint samples (tens of micrograms). The workflow was initially tested on a set of ten painting mock-ups prepared using different proteinaceous binders (egg yolk, animal glue) and a variety of pigments containing different cations, including red lake extracted from wool fibres (Table 1). The mock-ups were used to verify that proteins could be confidently identified in paints of different inorganic compositions. Two replicates of different sizes were analysed for each mock-up, creating two sample sets labelled as “small” and “large” (see Sect. 1.1 of Supplementary Information (SI)), representing the range of sample sizes that would ordinarily be collected for lipid and protein analysis, respectively, by gas chromatography coupled to mass spectrometry (GC–MS). The amount of sample required for GC–MS protein analysis often precludes its use on artworks. Additionally, amino acid quantification with GC–MS does not always achieve accurate identifications^32^. This study, instead, aimed at achieving the confident identification of proteins from smaller samples using tandem-MS palaeoproteomics. In addition, palaeoproteomics allows the identification of the biological species from which the detected proteins originate, and their molecular damage profile. Characterisation of the protein damage is often achieved by calculating the extent of spontaneous modifications affecting specific amino acids over large timescales. For example, deamidation, a spontaneous reaction occurring on asparagine and glutamine residues over time, has been previously observed at high levels in ancient proteinaceous materials^33–35^.

**Table 1:** Description of the complete set of analysed materials: mock-up paints, and samples collected from “The Virgin and Child with Saint John and an Angel” (NG275).

Mock-up paints					
Sample label	Layers	Binding medium	Pigment (main metal ion)	Year of preparation	Support
A	Paint layer	Chicken egg yolk	Lead white (Pb)	2005	Teflon
B	Paint layer	Chicken egg yolk	Malachite (Cu)	2005	Teflon
C	Paint layer	Chicken egg yolk	Calcite (Ca)	2005	Teflon
D	Paint layer	Chicken egg yolk	Synthetic iron oxide (Fe)	2005	Teflon
E	Paint layer	Rabbit skin glue	Lead white (Pb)	1978	Glass
F	Paint layer	Rabbit skin glue	Smalt (Co/K)	1978	Glass
G	Paint layer	Chicken egg yolk	Yellow ochre (Fe)	1933	Wood panel
	Ground layer	Rabbit skin glue	Gypsum (Ca)		
H	Paint layer	Linseed oil	Yellow ochre (Fe)	1933	Wood panel
	Ground layer	Rabbit skin glue	Gypsum (Ca)		
I	Paint layer	Linseed oil	Madder lake (Al)	2013	Glass
J	Paint layer	Egg yolk	Madder lake (Al)	2013	Glass

Historic samples					
Sample label	National gallery sample name	Sample location	Inorganic materials identified by FTIR and/or SEM–EDX		
1:BP	OS14	Blue drapery (paint layer)	Ultramarine and lead white (pigments); calcium sulphate dihydrate (ground layer); calcium carbonate and oxalate (surface crust)		
1:GL	OS15	Blue drapery (ground layer)	Calcium sulphate dihydrate (ground layer)		
2:YP	OS16	Yellow drapery (paint layer)	Lead tin yellow and earths (pigments); calcium oxalate and carbonate (with unidentified metal counterion; surface crust)		

**Figure 1:**
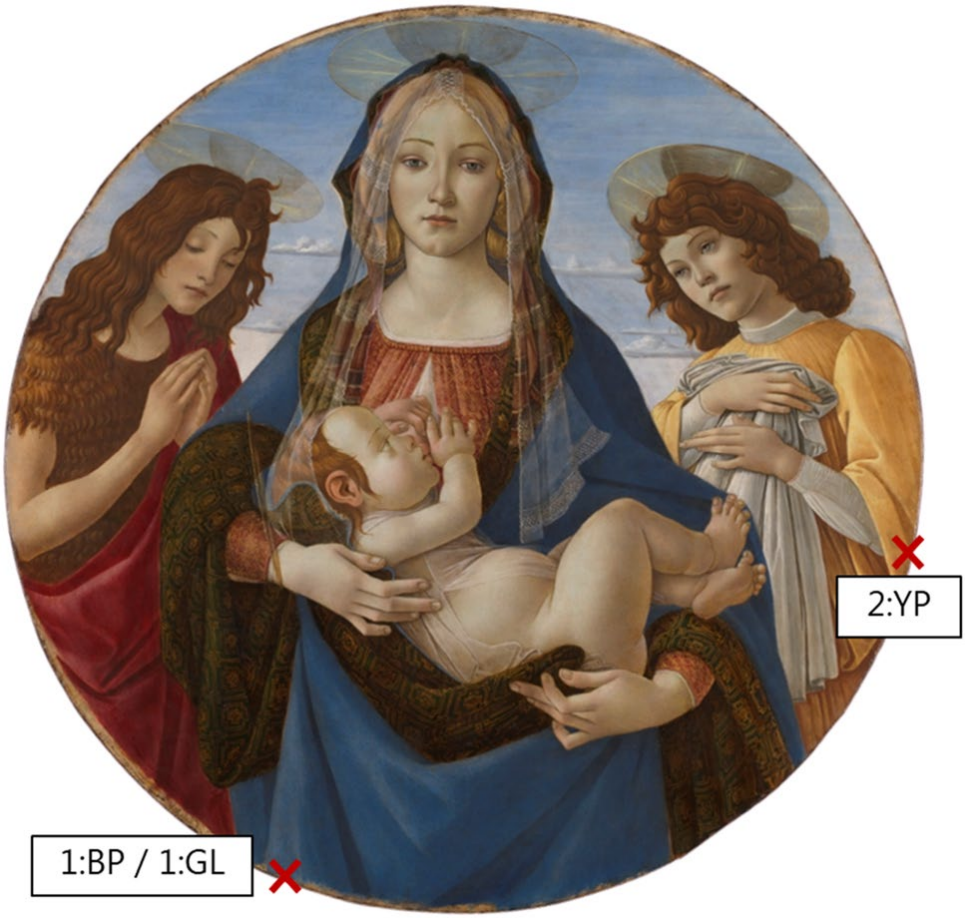
Workshop of Sandro Botticelli. The Virgin and Child with Saint John and an Angel. About 1490. Copyright: The National Gallery, London. The red crosses indicate the two spots where three samples were removed for palaeoproteomic analysis: 1:BP—blue paint of drapery; 1:GL—ground layer directly below sample 1:BP; 2:YP—yellow paint of garment.

After the mock-ups, the palaeoproteomic workflow was used to analyse three samples collected from a panel painting undergoing conservation treatment, “The Virgin and Child with Saint John and an Angel” (about 1490), attributed to the workshop of Sandro Botticelli (NG275, The National Gallery, London, UK). The samples were removed from areas containing different pigments (Table 1 and Fig. 1). The aim was to characterise the protein binder in the paint and ground layers detected by Fourier transform infrared spectroscopy, and to preliminarily investigate whether different pigments influence the protein damage pattern observed.

This work illustrates how proteomic analysis of pigmented micro-samples from artworks can provide detailed characterisation of the type, source, and damage status of the proteinaceous materials used. Although guidelines for the proteomic analysis of other cultural heritage materials have recently been published^36,37^, and a number of case studies applying proteomic approaches to paintings are available, a discussion of the proteomic analysis of paintings is, to the best of the authors’ knowledge, still lacking. Therefore, this work ultimately aims at providing heritage scientists with guidelines for the interpretation of protein-sequencing results from such objects.

## Results

### Mock-up samples

The analysis of samples from the 10 mock-ups prepared with different combinations of binders and pigments permitted the identification of the proteinaceous binder used, either animal glue or egg, for all of the large and in 8 of the 10 small samples. The number of the proteins, peptides and MS/MS spectra supporting the identifications is provided in Supplementary Table S1.

In four of the small and all of the large samples of paints bound with egg yolk, proteins from egg white were also identified, namely lysozyme, ovoinhibitor, ovoalbumin, and ovotransferrin. This is probably due to the manual separation of the tissues (yolk from white) during preparation of the binder, which often does not assure the complete separation of egg white proteins from the yolk. Thus, the detection of egg white proteins does not necessarily indicate the intentional use of whole egg, rather than just yolk. The taxonomic source of the egg yolk as *Gallus gallus* (chicken) was confidently determined for both sample sizes of all the mock-ups, based on the identification in each sample of at least one protein with species-specific peptides for chicken. The sequences of all the non-species-specific peptides identified still match the chicken sequence and can therefore be assigned to chicken by parsimony. Animal glue from *Oryctolagus cuniculus* (rabbit) was identified in all the large samples, and in 2 of the 4 small samples of the mock-ups where it is present as a binder of the paint or ground layer. In addition, type III collagen, which has been suggested as an indicator for hide glue^28,38^, was identified in paints E and F. These results agree with the known use of rabbit skin glue in these mock-ups (Table 1).

Mock-ups G and H were deliberately sampled to contain both paint and ground layers. For both mock-ups, animal glue from the ground layer was identified in the large, but not the small sample. Surprisingly, chicken vitellogenin-2 and ovalbumin were identified by a few peptides in the small and large samples of paint H, respectively. This mock-up was prepared in the 1930s and labelled as a linseed oil and yellow ochre paint on a gypsum in glue ground layer. The detection of a small number of egg peptides in both samples might suggest cross-contamination of the paints during the preparation, storage, or sampling, and illustrates how cautiously one must interpret results when only a small number of peptides or proteins are detected.

In paints I and J, containing madder lake pigments prepared from dyed wool (see Sect. 1.1 of SI), several non-human keratins, including sheep-specific peptides, were confidently identified (Supplementary Table S4). The identified proteins and the corresponding species-diagnostic peptides from the mock-ups are reported in Supplementary Tables S4 and S5, respectively. These results are discussed in more detail in Sect. 1 of SI.

### Samples from the historic painting

The two paint-containing samples from “*The Virgin and Child with Saint John and an Angel*”, 1:BP (Blue Paint) and 2:YP (Yellow Paint), showed very similar protein composition. Proteins associated with chicken egg yolk (vitellogenin-1, vitellogenin-2, apolipoprotein B) were confidently identified in both samples (Supplementary Table S6). Avian serum albumin, a protein present in egg yolk, but also in egg white and blood, was also identified in sample 2:YP. The identification of this protein was supported only by two non-species-specific peptides, preventing the exact identification of its species of origin. However, both the peptides are compatible with the corresponding chicken sequence, therefore this protein can also be assigned to chicken by parsimony.

Peptides from collagen alpha-1(I) and collagen alpha-2(I), assigned exclusively to both *Ovis aries* (sheep) and *Capra hircus* (goat), were identified in all samples, but none of them allowed discrimination between these two species. Collagens are the most abundant proteins in many body tissues, such as bone, skin, and tendons^39^, and their identification is likely due to the use of animal glue, traditionally used as an organic binder in the preparation layer of panel paintings^2^. The identified collagens did not allow discrimination of the tissue used to produce the glue, since only type I collagens, expressed in almost all connective tissues^40^, were identified in all the samples.

Proteins from egg yolk and animal glue were detected in both samples 1:BP and 1:GL (Ground Layer), collected in the same spot from the paint and ground layer, respectively. The percentage of peptides identified for each protein source per sample supports the use of egg yolk as the paint binder and animal glue as the binder in the preparation layer, in agreement with the most common recipes at the time^2^. The identification of both protein sources in the sample removed from the paint layer (1:BP) is probably due to the mechanical means by which the samples were obtained (i.e. via a scalpel), and to the thinness of the paint layer (a few µm), making it almost inevitable that some of the lower preparation layer would inadvertently be removed when scraping off the outer paint layer. The detection of gypsum, commonly mixed with animal glue in the ground/preparation layer, by FTIR microscopy (Table 1 and Supplementary Table S3) also shows that sample 1:BP contained some of the underlying layer. The identification of one egg protein in sample 1:GL might be due to partial removal also of the paint layer, or to penetration of the paint into the preparation layer during painting production.

The number of the proteins, peptides, and MS/MS spectra supporting these conclusions is provided in Table 2. The exhaustive list of non-contaminant proteins identified are reported in Supplementary Table S6, and the corresponding species-diagnostic peptides in Supplementary Table S7. All reported proteins were identified when matching the spectra against a reference database containing all the publicly available sequences for common proteinaceous paint binders (Sect. 5.4). The search against the SwissProt database, to identify other protein sources, did not lead to any further identifications.

**Table 2:** Summary of the protein identifications for the micro-samples collected from “The Virgin and Child with Saint John and an Angel” (NG275).

Sample	Identified proteins	Total identified peptides	Species-diagnostic peptides	Matching MS/MS spectra	Protein source	Taxonomic source
1:BP	3	52	13	93	Egg yolk	*Gallus gallus*
	2	39	3	72	Animal glue	*Ovis aries/Capra hircus*
1:GL	1	3	–	3	Egg yolk	Avian
	2	42	2	79	Animal glue	*Ovis aries/Capra hircus*
2:YP	4	143	30	305	Egg yolk	*Gallus gallus*
	2	17	1	25	Animal glue	*Ovis aries/Capra hircus*

**Figure 2:**
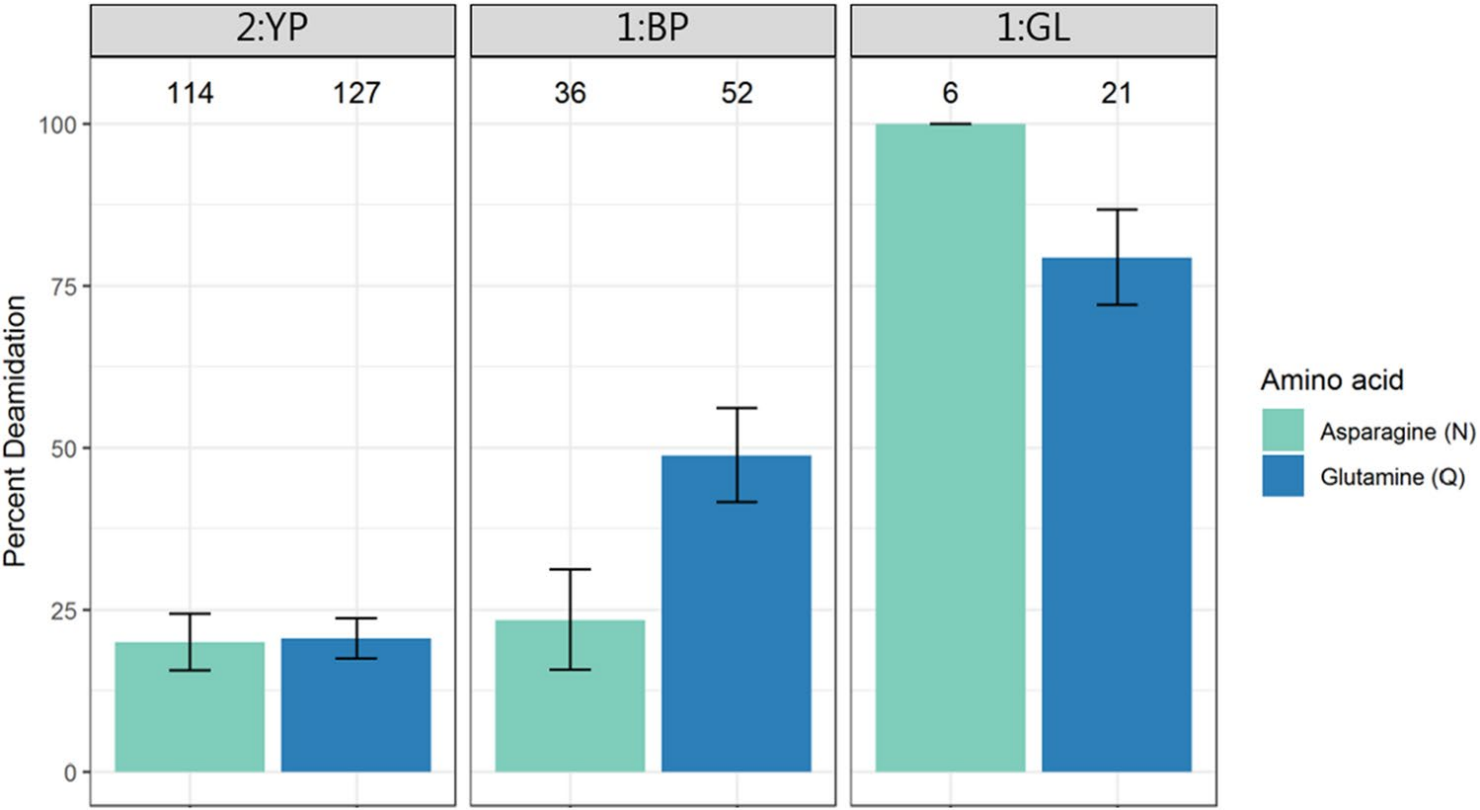
Percentage of deamidation of asparagine (N) and glutamine (Q) residues in the three analysed samples. Error bars indicate standard deviation around 1000 bootstrap replicates. Sample identifiers are shown at the very top, while the number of peptides used for the calculation is indicated above each bar.

**Figure 3:**
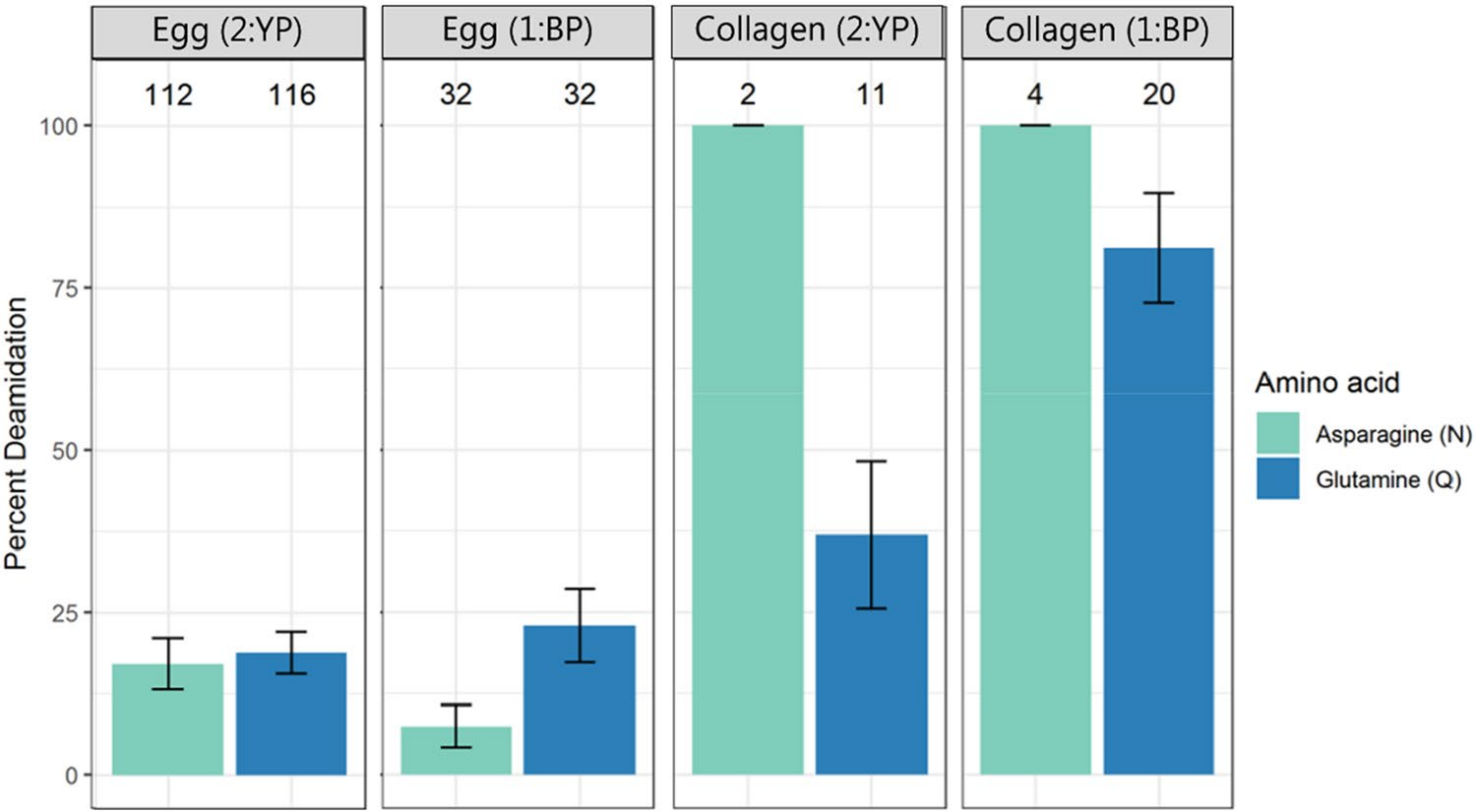
Percentage of deamidation of asparagine (N) and glutamine (Q) residues in egg proteins and collagen for the samples 1:BP and 2:YP. Error bars indicate standard deviation around 1000 bootstrap replicates. Sample identifier and protein class are shown at the very top, while the number of peptides used for the calculation is indicated above each bar.

The extent of deamidation was calculated and used to assess the damage of the proteinaceous materials (Fig. 2). Deamidation of asparagine was comparable in the two paint samples 1:BP and 2:YP, whereas the level of glutamine deamidation in the former was about two times as high as in the latter. For these samples, deamidation of both amino acid residues was lower than that of the ground layer sample 1:GL. Most of the peptides identified in this sample come from the collagens from animal glue, prepared by boiling animal remains for a long period of time, a process which is expected to increase protein deamidation^41^. In order to investigate the influence of the preparation process on protein damage, the deamidation levels of egg proteins and collagens were analysed separately for 1:BP and 2:YP, both containing more than one protein from each source. The plot reported in Fig. 3 shows that the degree of deamidation of collagens was higher than that of egg proteins for both samples. However, the calculations for collagens were based on no more than 20 peptides in either sample and for either amino acid, limiting the reliability of the calculation^42^.

The search for modifications associated with photo-oxidation^31^, a process exposed paintings are potentially very susceptible to, did not lead to significant results. The use of the MaxQuant Dependent Peptides algorithm also did not lead to the detection of other protein modifications.

## Discussion

The results obtained from the 10 mock-ups show that the adopted experimental protocol allows for the confident characterisation of proteins from painting micro-samples. Identification of the proteinaceous binder and its biological source was achieved for all the large samples. Starting from a similar sample size, other methods, such as amino acid analysis, might have allowed for the discrimination of the proteinaceous material, but not the identification of the source species and tissue. The confident identification of the proteinaceous binder and of its source in the majority of the small samples also shows that the sensitivity of the protocol can allow protein characterisation in samples even smaller than those routinely collected for other techniques.

The number of proteins and peptides detected varies throughout the set of mock-ups (Supplementary Table S1). It is not clear whether this is due to the variability of the amount sampled, as it was not possible to weigh the micro-samples or to calculate the binder-pigment ratios, or due to the presence of different pigments, which have been shown to affect protein identification using other techniques^24,25^. The data collected in this work were not sufficient to speculate on the factors influencing protein recovery, and further studies are needed to investigate the possible interference of the inorganic components on proteomic analysis.

Paints I and J contained madder lake pigment, and linseed oil and egg yolk as binders, respectively. The preparation of the lake pigment involved the extraction of the colourant from dyed sheep wool, using alkaline conditions at high temperature. These conditions favour peptide bond hydrolysis. Studies of painting samples containing pigments prepared in this way have shown the presence of protein residues within the pigment particles^43,44^. This indicates that some of the wool peptides are incorporated within the pigment and should therefore be detectable with proteomic analysis. Although keratins are often identified as contaminants in palaeoproteomics due to sample handling, non-human keratins were confidently identified in both sample sizes of I and J with peptides not matching the human sequences (Supplementary Table S5). These results show how the level of information retrieved via proteomics can, in some instances, even contribute to an understanding of pigment manufacture, and not only of the proteinaceous binder. The presence of these pigment-related proteins might have led to misidentifications if the analysis had been performed with a different technique, such as amino acid analysis via GC–MS, which is based on the quantitative analysis of amino acids in a sample compared to the profile of proteinaceous standard materials^45^. The presence of proteins from other sources than these standards will result in an unknown profile, likely leading to misidentifications or false positive results. This phenomenon has already been reported in literature concerning proteins from microorganisms present on the painting^32^.

The study of the mock-up paints proved that the adopted workflow, based on state-of-the-art mass spectrometry and an experimental approach optimised for ancient proteins, can lead to the confident characterisation of protein residues in micro-samples from paint layers. The application of this workflow to the set of three micro-samples removed from *The Virgin and Child* panel painting, attributed to the workshop of Sandro Botticelli, led to the identification of chicken vitellogenins and apolipoprotein in all paint samples, suggesting the use of egg tempera as paint binder. The use of whole egg, or egg white and yolk separately, as paint binder is well known^2^, and is reported in previous studies on paintings from Botticelli’s workshop^46^. The discrimination between whole egg, egg white, and yolk is theoretically possible based on the identification of tissue-specific proteins, such as vitellogenins and apolipoproteins^47^, but practically challenging, since manual separation of yolk and egg white does not guarantee the absence of protein contamination between the two tissues. Nonetheless, in the samples analysed, only proteins associated with egg yolk, and no egg white proteins, were identified, suggesting that egg yolk was exclusively used.

Collagens from sheep or goat were identified in all panel painting samples. Collagens are the main protein component of animal glue, a material extensively used in artworks^2^. The percentage of collagen peptides in the sample representative of the ground layer (1:GL) is higher than in the samples representative of the paint layer (1:BP and 2:YP) (Table 2), suggesting that animal glue was probably used as the binder in the ground layer.

The deamidation level of the proteins extracted from the paint layer samples 1:BP and 2:YP was comparable. In contrast, sample 1:GL, collected from the ground layer and containing mostly collagen, showed higher rates of deamidation (Fig. 2). This is probably due to the preparation of the animal glue, which requires prolonged boiling of animal remains. This process is expected to promote deamidation of the proteins, as temperature is one of the main factors influencing the kinetics of the reaction^48^. The significant influence of the preparation of such materials on protein damage has been previously observed^31^.

Interestingly, photo-related protein damage was not observed, despite the presence of pigments in the paint, commonly considered photo-sensitisers^31,49^, and the exposure of the artwork to light. The lack of damage might be due to factors protecting the paint, such as a frame, since all samples were removed from the edge of the painting, or the presence of a varnish layer. Nonetheless, the data collected are not sufficient to speculate on why no significant photo-damage was observed in the identified proteins, or why the different pigments in the two analysed paints did not cause different protein degradation patterns at a detectable level. The photo-oxidation level of the paint in other areas of the painting should be investigated, and further studies are needed to characterise protein photo-oxidation in a complex paint system, with a particular focus on the influence of the inorganic components.

In proteomic analyses of artworks, the identification of damage-related modifications is commonly limited to deamidation and photo-oxidation damage. However, the identification of other modifications might provide information about the preservation and display history of the object. A search for unspecified post-translational modifications (PTMs) was also performed on the samples from the painting, but no new modifications were detected at a significant level.

As proteomic analyses are becoming more and more common in heritage science, it has become apparent that a set of good-practice guidelines for the analysis of such results is required. Similar guidelines have recently been published for the palaeoproteomic analysis of other materials^36,37^. Advice contained in such guidelines can also be applied to the analysis of proteins in artworks, including avoiding the use of materials known to cause protein contamination, such as latex gloves^50^. Nonetheless, a set of guidelines tailored to the study of paintings and their specific challenges is beneficial. Therefore, the analysis of the three panel painting samples from above can be used to illustrate how to best identify and characterise the proteinaceous materials present in an artwork.

The identification of proteins in a sample is usually based on the comparison between experimental MS/MS spectra, and theoretical spectra generated by a software from the user-selected database, i.e. the protein sequences that the software will look for in the sample. The choice of the database is of paramount importance, since only the proteins present in the database will be found in the sample. Therefore, ideally, all the available protein sequences should be included. However, using very large databases (such as the TrEMBL database from Uniprot^51^) drastically increases the computing resources necessary to perform the search, and the probability of misidentifications. The identification of all the proteins present in the sample and the choice of the database can therefore become an iterative process. For the first search, any general knowledge on the possible sample composition should be used to build the smallest possible database of proteins. In the case of materials used in paintings, a search against a database containing only the most common proteins in artworks (egg, collagens, and milk proteins) is often sufficient. The samples should then be searched against a larger database to test for the presence of other proteins, and unexpected protein sources. Any additional hits are then included in a new, more limited, database. In this study, samples were searched against the most common proteinaceous paint binders first. Afterwards, they were searched against the Swiss-Prot database from Uniprot^51^. The second search did not lead to any further identification for the painting samples, whereas in two of the mock-up paints it allowed for the identification of non-human keratins, deriving from the wool peptides co-extracted with the madder lake (see Sect. 1.1 of SI). A third search was therefore performed on these samples with a database containing the publicly available sequences for sheep keratins, resulting in the identification of several species-specific sheep peptides (see Sect. 1.2 of SI).

In the creation of the theoretical spectra derived from the database, the software needs to know what peptides to search for, and thus how to “digest” proteins in silico. If the sample has been digested with an enzyme, the software will cut the protein sequences in the database following the enzyme specificity. If no enzymatic digestion is performed, or if there is reason to believe unspecific protein hydrolysis has occurred (e.g. because of advanced age and/or damage of the proteins), an unspecific search can be performed. In this case, the software will look for any possible peptide from the protein sequences in the database (limited by user-defined minimum and maximum length). However, like the use of a large database, this type of search will require more computing resources, and increase the probability of misidentifications. Therefore, an unspecific search is only recommended if the material is highly degraded, as can be evaluated by damage patterns like deamidation, and/or if the material is known (or suspected) to have been treated in harsh conditions. In this study, an unspecific search was performed on the mock-ups containing madder lake, where the presence of unspecifically-cleaved peptides was expected due to the pigment extraction from wool with a strong alkali at a high temperature (see Sect. 1.1 of SI). The unspecific search on these samples led to the identification of many short and non-enzymatically-cleaved peptides.

**Figure 4:**
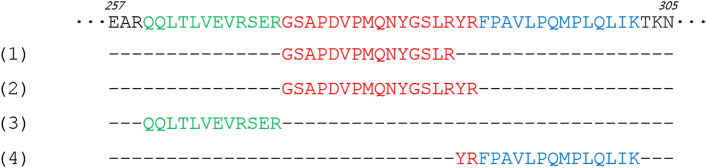
Portion of sequence of chicken vitellogenin-2 (P02845). The numbers on top of the sequence indicate the start and end positions of the shown peptide. The detection of only the tryptic peptides (1) and (2), in red, is not sufficient for protein identification, since the amino acidic sequence of peptide (1) is covered entirely by peptide (2). Peptides (1), (3), and (4) are non-overlapping, and therefore can be used to identify the protein. Peptides (2), (3), and (4) can be used for protein identification despite the partial overlapping of peptides (2) and (4), since neither entirely covers the sequence of the other.

The first step in the analysis of the results of every proteomic study is establishing which identifications can be considered confident and significant, and what must be discarded due to insufficient evidence. It is a common practice in palaeoproteomics to consider a protein as confidently identified only when at least two non-overlapping peptides unique to that protein have been identified^36,52^. In this case, “non-overlapping peptides” means that one of the peptides does not cover the amino acid sequence of the other entirely, and thus they must cover distinct sequence regions (Fig. 4). In addition, the identification of peptides is subjected to a scoring system, relying on the quality of the MS/MS spectra acquired for that peptide. In the MaxQuant software, the peptide score is calculated from several parameters, based on the comparison of the experimental spectrum with the theoretical spectra generated by the software from the protein database^53^. A score threshold is usually set during data analysis to exclude low-quality spectra that would have poor peptide identifications.

**Figure 5:**
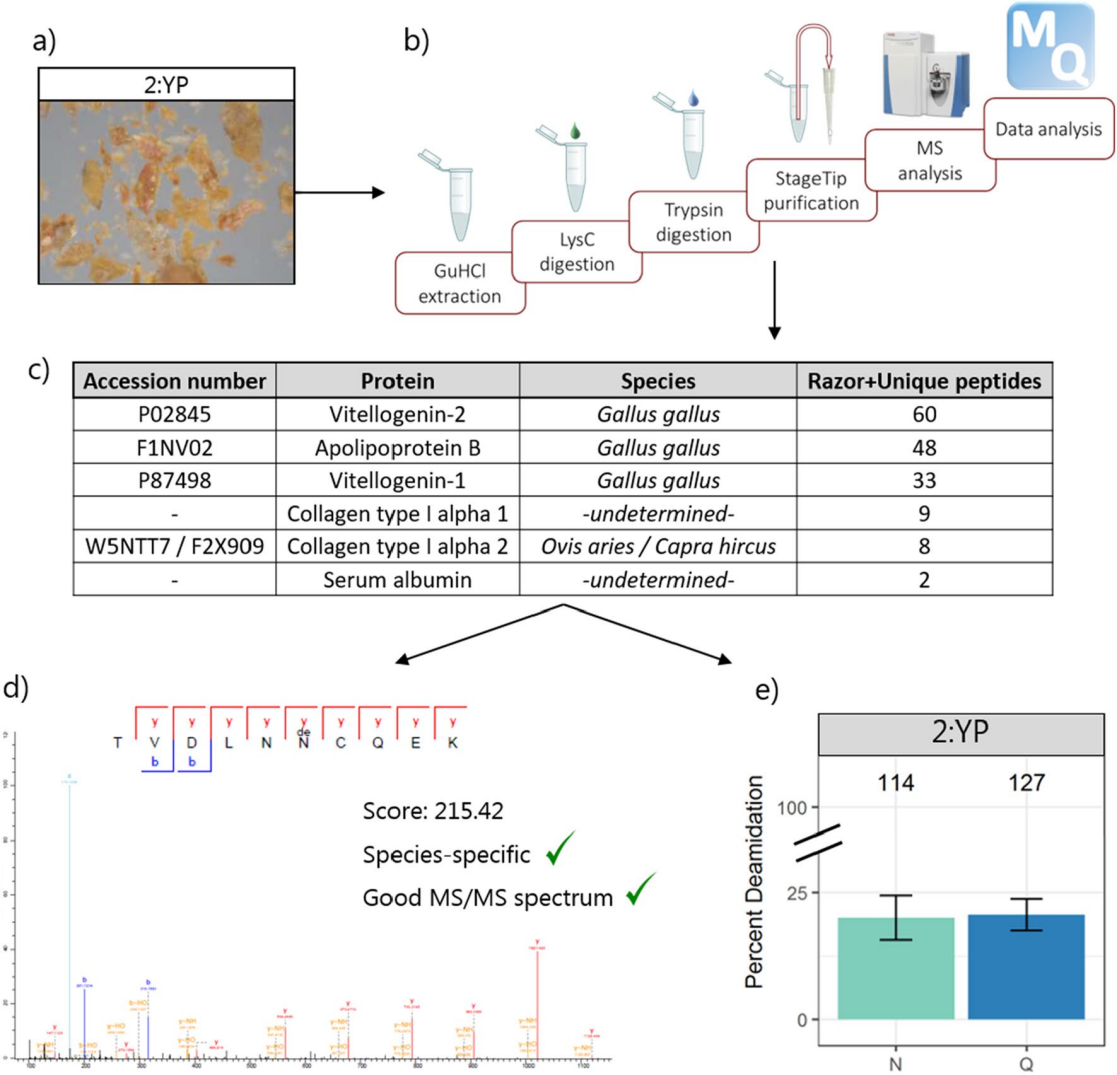
Scheme of the proteomic workflow for the analysis of a micro-sample from an artwork. After the sampling, the solid sample (**a**) is processed to extract and sequence proteins (**b**). The identification of the proteins present (**c**) and of their biological origin is based on the manual check of the MS/MS spectra: a good spectrum, identifying a species-specific peptide (**d**), allows species identification. Finally, the damage level of the proteins is investigated (panel **e** shows for example the percent deamidation level).

The determination of the taxonomic origin of a protein is based on the confident identification of at least one species-specific peptide, i.e. not matching with any other sequenced species, with the distinguishing amino acid(s) covered by at least one ion fragment. Since the database used for the search contains, in most cases, a limited number of proteins, the specificity of each peptide should be verified by comparing its sequence with all the publicly available protein sequences. This can easily be done via search engines such as the NCBI tool BLAST^54^, which will show all the proteins containing the query sequence, and the close matches. When assessing the specificity of the peptide, it should also be verified that no amino acid substitution might be the cause of a false-positive. Three cases in particular are very common: (1) leucine and isoleucine substitution: the two residues have different structures, but the exact same mass, and cannot be distinguished by tandem-MS analysis; (2) asparagine and aspartic acid, and glutamine and glutamic acid substitutions: discrimination is only possible if the non-deamidated form is confidently identified by at least one spectrum; (3) serine and alanine substitution: the difference between these two residues is an hydroxyl group on the serine, which can be added by oxidation to other amino acids, namely proline and methionine. Therefore, serine and alanine close to an oxidized residue can only be distinguished if a fragment ion separating the bond between the two residues allows for the localisation of the hydroxyl group. For these reasons, a manual check of all the identified MS/MS spectra is strongly encouraged (Fig. 5).

A peptide can be considered species-diagnostic also when matching with more than one species if only one of them is likely to have been used to produce the material. This is based on the historical and geographical provenance of the object. For example, the peptide TGPPGPAGISGPPGPPGPAGK of collagen alpha-2(I), detected in sample 2:YP, has been considered diagnostic for either sheep or goat despite it matching two more species: Tibetan antelope and water buffalo, both indigenous to Eastern Asia (Supplementary Table S7). The use of animal glue produced from these species is unlikely for an object painted in Italy in the late fifteenth century.

Species identification is easier when proteins with less conserved sequences, i.e. with high inter-species sequence variability, are detected. Proteins with low variability often only allow the identification of a restricted group of species at best, unless sequence coverage allows the recovery of at least one of the few species-specific peptides^55^. In this work, the taxonomical origin of collagen could only be limited to either sheep or goat, which are genetically very close and, therefore, have generally very similar protein sequences.

One of the most powerful applications of protein sequencing is the ability to identify and localise modifications on a specific amino acid of a protein sequence. Most proteomics data analysis software allow searching for several protein modifications. These modifications can not only increase the peptide rate identification, by allowing for the detection of modified peptides that would have otherwise been unidentified, but are also used for the investigation of the damage status of the proteins. Deamidation of asparagine and glutamine residues is one of the most studied PTMs in palaeoproteomics. High levels of this modification, spontaneously occurring over time, have been observed in very ancient samples, leading to several studies using it as an indicator of protein damage (see for example^27,56^) and to discriminate original protein content from modern contamination^57^. However, the rate of the reaction is highly affected by environmental factors and the protein sequence^58,59^. Gathering as much information as possible about the ageing conditions of the studied material is fundamental before any interpretation of the deamidation level can be done, and the comparison with a modern standard material of similar composition should be included whenever possible. Such a comparison also helps in establishing whether the adopted protocol causes the introduction of artificial modifications, and therefore if the damage pattern observed in samples from artworks is authentic, or due to artefacts produced during sample treatment. In this work, reference materials containing the same pigments as the analysed samples from the panel painting were not available. However, the level of deamidation of egg proteins from samples 1:BP and 2:YP was compared to that of mock-ups A-D, containing a range of pigments bound with egg, to verify whether the damage observed was to be attributed to the sample treatment protocol rather than to authentic damage of the proteins. In all cases, the deamidation level of the samples from the panel painting was higher than that of the mock-up samples. The results obtained in this work, as well as in previous palaeoproteomic studies^31^, also highlight the importance of considering preparation procedures of the original proteinaceous material when assessing protein damage. For example, when collagens are identified, as in the samples from *The Virgin and Child*, the well-reported preparation procedures of glue, which include prolonged boiling of the raw material, must be taken into consideration. The harsh extraction conditions in the manufacture of madder lake pigment is also an example of knowledge to be taken into account when assessing protein damage on sheep keratins found associated with this pigment.

Generally, the more modifications that are searched for, the larger the computational space required. Moreover, it is virtually impossible to define all the modifications present in damaged proteins, such as those found in ancient materials. The number of investigated protein modifications could be increased by detecting mass shifts between unidentified MS/MS spectra and the spectra of identified peptides (usually unmodified). This approach has previously led to the identification of modifications related to photo-damage and exposure of a wall painting to light^31^. The significance of the newly detected modifications should be evaluated based on the feasibility of the chemical process leading to the modification, and on the number of occurrences in each sample. The identification can be further confirmed by comparison with standard materials, and by the occurrence of the same modification in comparable conditions from literature.

## Conclusions

The results reported in this work show that confident protein characterisation can be achieved from quantities as low as tens of micrograms of pigmented material removed from paintings, even in samples representing a single layer and in the presence of inorganic pigments. The use of state-of-the-art mass spectrometry technology, coupled with a workflow optimised for analysis of ancient protein residues, allows for the determination of the type of proteinaceous material used, and also its biological source thanks to the possibility to discriminate between species, and even tissues. Proteomic results from the different layers, supported by knowledge of artistic techniques, can help to reconstruct the use of different materials in the stratigraphy of the painting. After successfully testing on a series of mock-up paints containing proteinaceous binders, the adopted approach revealed the use of chicken egg yolk as the paint binder and animal glue from sheep/goat as the binder of the ground layer in two sampling locations of the panel painting “The Virgin and Child with Saint John and an Angel”, attributed to the workshop of Sandro Botticelli. The evaluation of the protein damage suggested that most of the damage is due to the preparation procedures of the original materials, and that the different pigments in the two sampling locations do not appear to have significantly influenced the protein damage pattern. Further studies are needed to investigate photo-related and pigment-specific damage in proteins in a paint system, given the presence of high proportions of inorganic pigments within such samples.

Although proteomics has been used in the study of artworks for over 15 years, its application in this field remains limited. A guide to the proteomic analysis of painting micro-samples has been given in this work, which aims at increasing the accessibility of this approach to heritage scientists, and hopes to inspire further application of this powerful technique. In the future, proteomics should be exploited more to connect the detailed characterisation of protein-based materials with the original production and the preservation history of artworks. Furthermore, the intrinsic untargeted nature of this technique, as opposed to techniques based on the comparison with a small number of standards, also allows for the potential identification of possibly unexpected protein-containing materials.

## Methods

### Samples

20 samples from 10 paint mock-ups were analysed to test the protein extraction protocol on paint materials, prior to the analysis of the historical samples. The details of these samples are reported in Supplementary Information.

Three micro-samples were removed from the panel painting “The Virgin and Child with Saint John and an Angel” by Sandro Botticelli’s workshop (Acc. No. NG275, The National Gallery, London, UK). The size and the nature of the samples, collected in a fine powder and barely visible to the naked eye, did not allow accurate measurement of their weight. Based on the experience of the authors with samples of similar nature, which are typically 20–300 μg in size^60^, all samples were estimated to be in the range of 10–20 μg, although the sample weight also depends on the pigment present. All samples were removed from the edge of the painting, near areas of existing damage, and specifically from areas where no visible repainting was present. In one location, it was attempted to collect the paint and the ground layer beneath separately, although the flaky properties of the materials and the thinness of the layers did not allow a perfect separation. Nonetheless, samples 1:BP and 1:GL are believed to mostly consist of the paint layer and the ground layer, respectively (Table 1). Sample 2:YP was collected from the paint layer in a different location (Fig. 1).

### Protein extraction protocol

Samples were treated with a protocol previously described^31^. In order to perform protein extraction and digestion, the micro-samples were placed in separate Protein Lo-Bind tubes (Eppendorf, Germany) and, along with a blank control, incubated for 2 h at 80 °C with 100 μL of an aqueous buffer containing: 2 M guanidinium chloride (GuHCl), 10 mM tris(2-carboxyethyl)phosphine (TCEP), 20 mM chloroacetamide (CAA), and 100 mM trisaminomethane (Tris). The pH was adjusted to around 8.0 using NH_4_OH 10% when needed. Proteins were digested under agitation for 2 h at 37 °C in-solution with 0.2 μg rLysC (Promega, Sweden). Samples were then diluted to a final concentration of 0.6 M GuHCl using 25 mM Tris in 10% acetonitrile (ACN) in water, and digested overnight under agitation at 37 °C with 0.8 μg Trypsin (Promega, Sweden). Samples were then acidified to pH 2 using 10% trifluoroacetic acid (TFA) to quench digestion. The resulting peptides were collected on in-house made C18 extraction stage-tips, as previously described^50^. Stage-tips were stored at − 18 °C until mass spectrometry analysis.

### nLC-MS/MS

The nLC-MS/MS method used for the mock-up samples is reported in the Supplementary Information.

For the painting samples, the extracted peptides were eluted from the stage-tips using 30 μL 40% ACN, 0.1% TFA in water. Extracts were placed in a vacuum centrifuge at 40 °C until approximately 3 μL of solution were left, and then rehydrated with 5 μL of 0.1% TFA, 5% ACN. The peptides were then separated on a 15 cm column (75 μm inner diameter) in-house laser pulled and packed with 1.9 μm C18 beads (Dr. Maisch, Germany) on an EASY-nLC 1200 (Proxeon, Denmark) connected to a Q-Exactive HF-X (Thermo Scientific, Germany) on a 77 min gradient. Buffer A was 0.1% formic acid in milliQ water; buffer B was 80% ACN and 0.1% formic acid. The peptides were separated with increasing buffer B, going from 5 to 30% in 50 min, 30–45% in 10 min, 45–80% in 2 min, held at 80% for 5 min before dropping back down to 5% in 5 min and held for 5 min. Flow rate was 250 nL/min. The column temperature was maintained at 40 °C using an integrated column oven. A wash-blank method using 0.1% TFA, 5% ACN was run in between each sample to hinder cross-contamination. The chromatograms of the large sample from mock-up C and the 2:YP sample from *The Virgin and Child* are reported in Supplementary Fig. S5 as examples.

The mass spectrometer was operated in data dependent top 10 mode. Spray voltage was 2 kV, S-lens RF level at 50, and the heated capillary kept at 275 °C. Full scan mass spectra were recorded at a resolution of 120,000 at m/z 200 over the m/z range 350–1400 with a target value of 3e6 and a maximum injection time of 25 ms. HCD-generated product ions were recorded with a maximum ion injection time set to 118 ms. The target value was 2e5 and spectra were recorded at a resolution of 60,000. Normalised collision energy was set at 28% and the isolation window was 1.2 m/z with the dynamic exclusion set to 20 s.

### Data analysis

The MS/MS spectra were identified with the MaxQuant software^61^ (version 1.6.1.0), matching them against a reference database containing all the publicly available sequences for the most common proteinaceous paint binders (collagens, egg proteins, milk proteins). In order to investigate the presence of protein residues originating from additional sources, the spectra were then matched against the SwissProt database (downloaded January 2017)^51^, which contains a large selection of manually reviewed protein sequences from a wide variety of species. The software was set to search for tryptic peptides with up to a maximum of 5 modifications per peptide. All samples were matched against the database of paint binders twice, with different variable modifications. The first search included the following variable modifications: oxidation of methionine, deamidation of asparagine and glutamine, conversion of N-terminal glutamine to pyroglutamic acid, conversion of N-terminal glutamic to pyroglutamic acid, and hydroxyproline. A second search was performed with the following variable modifications, including PTMs identified as markers of photo-oxidation^31^: acetylation of protein N-terminal, oxidation of histidine and tryptophan, di-oxidation of histidine and tryptophan, conversion of histidine to aspartic acid, conversion of histidine to hydroxyglutamic acid, conversion of histidine to glycine, oxidation of tryptophan to kynurenine, oxidation of tryptophan to hydroxykynurenine, and hydroxyproline. Carbamidomethylation was set as a fixed modification for all searches. The minimum peptide length was set to 7, with up to two missed cleavages. The false discovery rate (FDR) was set to 0.01 and the minimum score for unmodified and modified peptides was set to 40. The error tolerance was set to 5 ppm for the precursor and to 20 ppm for the fragment ions. The dependent peptides algorithm was applied in the first run, with an FDR of 0.01. Contaminant proteins were assessed using the contamination.fasta provided by MaxQuant, containing common laboratory contaminants (http://www.coxdocs.org/doku.php?id=maxquant:start_downloads.htm), including: primate keratins (likely from the laboratory space or through human handling of samples), excess trypsin, and Bovine Serum Albumin (a common laboratory reagent). Peptides assigned to contaminant proteins were filtered out and not considered further.

Proteins are considered confidently identified if at least two unique non-overlapping peptides are observed, unless otherwise specified. Peptides were considered species-diagnostic when, after search against the entire nrNCBI protein database via the BLAST alignment tool^62^, they were assigned to a single species, or to a limited number of species among which only one can be considered plausible, based on: (1) the nature of the samples, (2) the geographic origin of the sample, and (3) the dating of the sample.

The deamidation level was calculated using the deamidation tool previously described^31^ and freely available on GitHub (https://github.com/dblyon/deamidation). The Dependent Peptides algorithm of MaxQuant was used to search for unspecified protein modifications by comparing the MS/MS spectra of identified peptides (usually unmodified) to unidentified spectra^63^.

## Supplementary Information

Supplementary Information.

Supplementary Tables.

Supplementary Tables.

## Data Availability

The mass spectrometry proteomics data have been deposited to the ProteomeXchange Consortium (http://proteomecentral.proteomexchange.org) via the PRIDE partner repository^64^ with the dataset identifier PXD024397.
