# Supplementary material for: Palaeoproteomics guidelines to identify proteinaceous binders in artworks following the study of a 15th-century painting by Sandro Botticelli’s workshop
Source: Sci Rep. 2022 Jun 23;12:10638. doi: 10.1038/s41598-022-14109-w (PMC9226190; doi:10.1038/s41598-022-14109-w)
Supplement: Supplementary file 1 — Supplementary Information. [file 41598_2022_14109_MOESM1_ESM.pdf]

SUPPLEMENTARY INFORMATION

Palaeoproteomics guidelines to identify proteinaceous binders in artworks following the study of a 15th-century painting by Sandro Botticelli's workshop

F. Di Gianvincenzo[1]*, D. Peggie[2], M. Mackie[1,3], C. Granzotto[1,4], C. Higgitt[2], E. Cappellini[1]*

[1] Globe Institute, University of Copenhagen (Copenhagen, Denmark)
[2] National Gallery Scientific Department (London, United Kingdom)
[3] Novo Nordisk Foundation Center for Protein Research, University of Copenhagen (Copenhagen, Denmark)
[4] Art Institute of Chicago Department of Conservation and Science (Chicago, Illinois)
* Corresponding authors

1. Mock-up samples

1.1. Materials and methods

Ten mock-ups (A-J) produced for a variety of experimental purposes over the past 80 years and now held in the reference archive at the National Gallery, London (see Table 1 in the main text) were studied to test the analytical protocol on selected paint systems. Mock-ups A-D were reconstructions prepared at the National Gallery in 2005 using egg yolk as a binder and some common historical pigments: lead white, malachite, chalk, and iron oxide, respectively. This allowed the analytical protocol to be tested with paints based on different metal-containing pigments. Mock-ups E and F were reconstructions prepared at the National Gallery in 1978 using rabbit skin glue as a binder, and lead white and smalt pigments, respectively, providing slightly older paint samples containing a different proteinaceous binder. Mock-ups G and H were part of the Fogg reference set, prepared at the Fogg Museum, Harvard in 1933. These reconstructions were composed of two layers: a preparation layer containing rabbit skin glue and gypsum, followed by a paint layer containing yellow ochre bound with egg yolk and oil, respectively. The samples in each case contained material from both layers, testing the ability of the protocol to find proteins from different sources in the same sample. Finally, mock-ups I and J were reconstructions prepared at the National Gallery in 2013 with paint containing a madder lake pigment bound in linseed oil and egg yolk, respectively. The preparation of the lake pigment involves the extraction of a red colourant from dyed sheep wool using alkali conditions at high temperature. It has been shown *via* infrared spectroscopy that the preparation of lake pigments in this way results in the co-precipitation of the wool protein with the pigment[1,2], thus it was anticipated that evidence for the presence of sheep keratins might be observed in these samples. Since the protein of interest in this case is not associated with the binder, but with the pigment itself, and is therefore likely to be present in smaller amounts than the

binder, samples from mock-ups I and J were slightly larger than the others to maximise the chance of observing sheep keratins.

The paint samples were removed using a scalpel and two sample sizes, “small” and “large”, were collected and processed separately. It should be noted that the sizes of the samples in the two sets were estimated by eye. Paint samples are typically between 20 – 300 µg in size (see for example[3]). The small sample was estimated by eye to be at the lower end of this range, while the larger sample was approximately three times the amount of the smaller sample. In addition, the protein content in each sample will differ depending on the pigment/binder ratio of the paint. This ratio is not known since the paints were prepared following a traditional approach of adding the binder to the pigment in increasing amounts until the mixture reached the desired consistency. The smaller of the two samples was estimated to be close to the amount that would normally be sampled for lipid analysis by gas chromatography-mass spectrometry (GC-MS), while the larger of the samples was estimated to be an amount required for reliable results for amino acid analysis by GC-MS. Once confident protein identification was achieved using the larger of the samples, which also allowed checking for any adverse effects caused by the influence of the pigment on extraction and/or analysis, the capability of the protocol to identify proteins in the smaller samples was then assessed.

The samples from the mock-ups were processed following a protocol similar to the one described in the main text (Section 5.2 – 5.3), with two main differences: the extracted peptides were eluted from StageTips using: 20 µL 40% ACN, 0.1% TFA in water, followed by 10 µL 60% ACN, 0.1% TFA in water into a 96-well MS plate. The two elution solutions were merged to form one unique sample for injection. The MS analysis was performed on a Q-Exactive HF (Thermo Scientific, Bremen, Germany), operated in the same conditions as the Q-Exactive HF-X (see main text, Section 2.4) but with a maximum MS/MS ion injection time set to 108 ms.

The data analysis for these samples consisted of two MaxQuant runs for each sample. The experimental MS/MS spectra were first matched against a reference database containing all the publicly available sequences for the most common proteinaceous paint binders (collagens, egg proteins, milk proteins). The second MaxQuant run matched the spectra against the SwissProt database (downloaded January 2017)[4]. For both samples of mock-ups I and J, two more searches were performed against a database containing keratins from *Ovis aries*, searching for tryptic peptides in the first search and for unspecific peptides in the second one. All the unspecified parameters were the same for all searches, as described in Section 5.4 of the main text.

1.2. Results and Discussion

1.2.1. Protein identifications

The first objective of the analysis of the mock-ups was to verify if the experimental protocol allowed for the confident characterisation of protein residues extracted from a paint matrix. The source material and the taxonomic origin of the proteins was confidently identified in all

cases except for the glue layer of the two small samples of mock-ups G and H (Supplementary Table S1). For all the other samples, the origin of egg yolk and animal glue was identified at species level due to the identification of species-specific peptides for several binder proteins. Proteins derived from the same material (e.g., egg or animal glue) in a sample likely all come from the same species, as long as there is no evidence to the contrary. Therefore, following the parsimony principle, proteins without species-specific peptides are assigned to the same species when all unspecific peptides are compatible with that species identification (in the mock-ups, *Gallus gallus* for egg proteins, *Oryctolagus cuniculus* for collagens, and *Ovis aries* for non-human keratins).

The identification of proteins not present in the original composition of the paint, i.e. egg proteins in mock-up H, might be due to a carryover of peptides from other samples during the chromatography. Carryover contamination was not observed for the other samples. In addition, since egg proteins were identified in both samples of mock-up H, the contamination might have occurred during the preparation, storage or sampling of the mock-up paint films. Nonetheless, following this result, harsher washing protocols with a steep gradient of the organic eluent have been implemented between chromatographic runs.

Supplementary Table S1 reports a summary of the proteins identified in the mock-ups. Supplementary Table S4 (in a separate Excel SI file) reports in detail the list of proteins identified in the mock-ups, including keratins identified in mock-ups I and J matching the raw data against the SwissProt database. Keratins, primarily from humans, are the most common protein contaminants in laboratory environments, but their presence can also be due to object and/or sample handling, making contamination of keratins almost unavoidable. Nonetheless, several non-human keratins were identified in the samples containing madder lake (mock-ups I and J), probably as a result of the extraction of the dye from sheep wool, as shown by the presence of peptides not matching to human proteins (Supplementary Table S5, in a separate Excel SI file). The identified peptides also allowed for the identification of sheep (*Ovis aries*) as the taxonomic origin of the wool. In particular, in the large sample of mock-up I, a peptide with sequence matching only sheep and goat (*Capra hircus*) was identified, together with several peptides not matching the goat sequence; therefore, sheep is the only species for which the simultaneous identification of these peptides is possible. In general, for most of the wool proteins the species was identified on the basis of the exclusion of other species deemed unlikely because they do not produce wool, like red deer (*Cervus elaphus hippelaphus*). The identification of the species of origin of keratins was made easier by the underlying knowledge that any animal keratin identified in these samples was probably coming from the extraction of the colourant from the wool. In the case of an unknown sample, the interpretation of results with unspecific peptides would have been more ambiguous without further evidence.

The harsh treatment used for the extraction of madder colourants from dyed wool during the preparation of the pigment (strong alkali conditions at high temperatures) is very likely to cause the partial hydrolysis of the keratins from the wool. In order to verify the presence of peptides formed during this process, the samples from mock-ups I and J were searched against a database of sheep keratin sequences, first setting the software to search for tryptic peptides, and in a second run for unspecifically-cleaved peptides. The large sample of mock-

up J will be here discussed as an example of the obtained results. In both searches, no less than 6 species-specific sheep keratins were identified, highlighting the importance of the choice of database in the data analysis, as only one peptide specific for sheep/goat was found in the original Swiss-Prot search (see discussion about databases in Section 3 in the main text). In the unspecific cleavage search, the number of identified peptides was indeed higher than in the tryptic-specific search, showing that spontaneous hydrolysis had occurred, probably during the preparation of the lake pigment. However, the aim of the analysis of these samples, that is, the identification of ovicaprid and non-human keratins co-extracted with the madder lake and detectable in the paint, had already been achieved with the run against the SwissProt database. Therefore, detailed results from further searches are not reported, as in this particular case further characterisation of the keratins is not necessary. Nonetheless, readers can find the results of these MaxQuant searches in the PRIDE entry connected to this work.

*Supplementary Table S1 - Summary of the confidently identified proteins extracted from the mock-ups. *This material possibly derives from contamination during the preparation, storage or sampling of the mock-ups.*

Mock-up	Protein source	Taxonomic source	Sample size	Total proteins	Total peptides	MS/MS spectra
**A**	Egg yolk	*Gallus gallus*	Small	2	8	9
			Large	6	196	305
**B**	Egg yolk	*Gallus gallus*	Small	4	160	231
			Large	7	335	509
**C**	Egg yolk	*Gallus gallus*	Small	3	44	56
			Large	8	180	301
**D**	Egg yolk	*Gallus gallus*	Small	7	203	378
			Large	7	266	496
**E**	Animal glue	*Oryctolagus cuniculus*	Small	3	70	219
			Large	3	171	623
**F**	Animal glue	*Oryctolagus cuniculus*	Small	3	19	38
			Large	3	54	139
**G**	Animal glue	*Oryctolagus cuniculus*	Small	-	-	-
			Large	2	7	6
	Egg yolk	*Gallus gallus*	Small	7	276	598
			Large	10	480	1406
**H**	Animal glue	*Oryctolagus cuniculus*	Small	-	-	-
			Large	1	5	9
	Egg yolk*	*Gallus gallus*	Small	1	10	11
			Large	1	2	3
**I**	Wool	*Ovis aries*	Small	5	51	664
			Large	6	72	1021
**J**	Egg yolk	*Gallus gallus*	Small	13	780	2641
			Large	15	827	2939
	Wool	*Ovis aries*	Small	4	57	479
			Large	4	56	547

The identification of keratins from wool provides valuable information about pigment manufacture, but the presence of these proteins could be misleading when protein analysis is performed with techniques other than proteomics, or if proteomics approaches were used without knowledge of the pigments present in the sample. One of the most common techniques for the investigation of proteins in paints is GC-MS, based on the quantitative analysis of amino acids in a sample compared to the relative amounts within proteinaceous material standards. The presence of unexpected protein-based materials will give an unknown amino acidic profile, as mentioned in previous literature[5]. Colombini, et al. [5] also mentions that the presence of fungi and bacteria might interfere with the identification of the original material, since the co-extraction of proteins from these organisms is possible. Using these protocols, the unknown amino acidic profile might be forced into the profile of one of the standard materials during the statistical analysis of the GC-MS results. The analysis of paint samples with proteomics overcomes this problem by allowing the confident identification of all the proteins present, even from materials that are not expected or are considered unconventional.

The samples chosen to test the protocol were selected in order to include a range of different pigments. The presence of certain metal species derived from pigments has been shown to influence protein analysis approaches in paint samples[5-7], but the influence of pigments on a proteomics protocol has never been investigated. The results obtained in the current study, evaluated in terms of number of total peptides identified in each sample, show some variability. However, this appears to be related to the sample size (and amount of protein in the sample) rather than to any influence of the specific pigment, which could not be investigated in detail since samples were not analysed in replicates and quantification was not performed. A qualitative evaluation of the results shows that the presence of the different pigments selected did not hinder the confident characterisation of the protein residues in any of the samples. Therefore, the implementation of a clean-up step, occasionally included in sample treatment protocols for protein analysis to remove pigments[6,8,9], is not necessary and might, on the contrary, cause loss of peptides.

As discussed in Section 1.1, the protein content in each sample will differ depending on the pigment-binder ratio of the paint. This is related to the specific pigment used, how the paint was made, and the amount of sample analysed. Neither the pigment-binder ratio nor the weight of sample were measured in these experiments, and therefore the size of the samples can be considered only approximately constant throughout the “small” and “large” sample sets. In particular, both samples of mock-ups I and J were intentionally larger than the others in the respective sample sets, in order to increase the chances of detecting wool peptides. This is reflected in the high number of egg peptides identified in both samples of mock-up J (Supplementary Figure S1 and Supplementary Table S1). The pigment/binder ratio and the sample size are probably the primary causes of the variation of the number of total peptides identified in the mock-ups (Supplementary Figure S1).

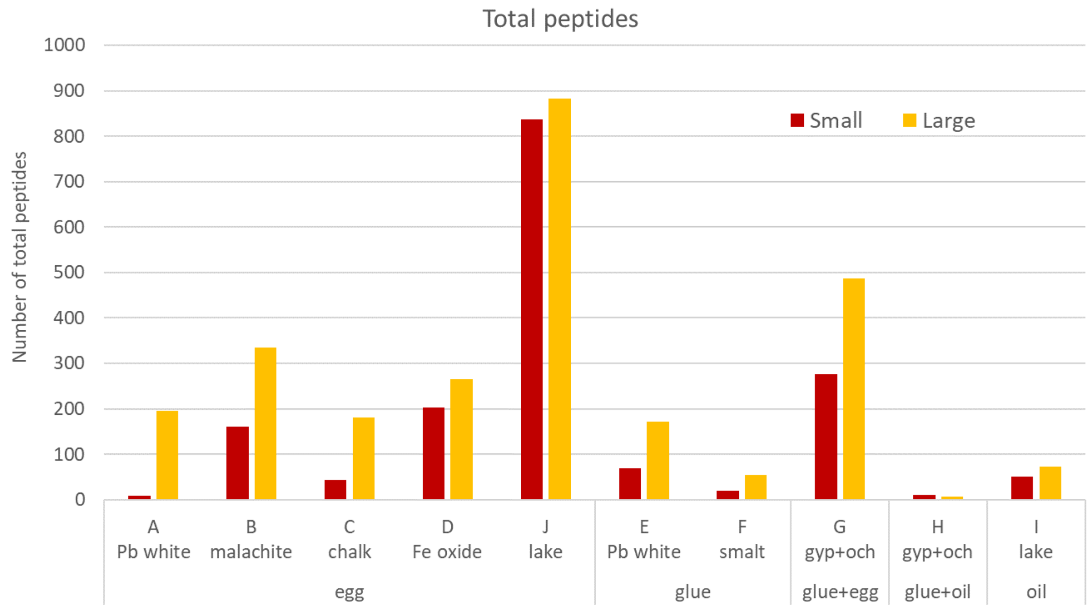

Supplementary Figure S1 - Total number of peptides identified in the mock-ups, grouped by paint binder.

Since the paint reconstructions were prepared in different years, the age of the paint might also be a factor affecting the degradation of proteins, and therefore the amount of protein extracted. However, no pattern of correlation between the age of the mock-ups and protein recovery was observed. On the contrary, the number of peptides identified in both samples of mock-up G was the second highest of each sample set, despite G being prepared up to 80 years before the other paints (see Table 1 in the main text). This might suggest that, in this case, the difference in age-related protein damage across the sample set was not a significant influence on protein recovery but systematic assessment of the impact of age within samples of a single pigment was not undertaken. It should also be noted that these mock-ups have also had a relatively short ageing period compared to Old Master paintings such as the 15th-century painting also studied in this work. Further, these mock-ups have only had limited light exposure having remained within the laboratory environment.

Although the presence of pigments did not appear as a significant influence on protein analysis in this work, dedicated studies should be performed to assess if any metal species, and in particular those derived from pigments, influence the proteomic characterisation of protein residues extracted from artworks and paintings.

1.2.2. Protein damage

The calculated levels of deamidation for the mock-ups are reported in Supplementary Table S2 and Supplementary Figure S2. Only the data relative to the large samples are reported in the graph, since the deamidation levels are comparable between the two sizes. The deamidation level was not considered reliable when less than 20 peptides could be used for the calculations, based on similar occurrences in recent literature[10].

The level of deamidation has often been regarded as an indicator of the ageing of ancient proteins[11], and can be used in cultural heritage studies to distinguish original proteins and

modern contaminants (Ramsøe, et al. [12] and literature therein) by comparing the results from the sample with a standard material of similar composition. However, as previously observed for materials used in artworks[13] and discussed in Section 3 of the main text, a significant source of deamidation is likely to be the processing of collagen to make animal glue[14]. In the mock-up samples, this is shown by the difference in the damage of egg proteins in mock-ups A-J (average level: 6% for asparagine (N), 1% for glutamine (Q)) compared to the collagen in mock-ups E-F (average level: 56% for N, 2% for Q) (compare to Figure 2 in the main text).

Although the major cause of collagen deamidation is probably from the preparation of the glue, a limited investigation into the effects of ageing on other proteins was possible, since the reconstructions examined in this study were made at different times. As noted above, all of the samples are relatively 'young' and have had limited light exposure and the pigments present varies between the samples, making it difficult to drawn firm conclusions. That said, mock-ups G and J both contained paint bound with egg yolk, for example, but were prepared in 1933 and 2013 respectively. In addition, some animal glue proteins (collagen) from the preparation layer were also present in mock-up G, while wool proteins (keratin) derived from the lake pigment were present in mock-up J. The comparison of the deamidation levels of the different protein sources in both samples is shown in Supplementary Figure S3, which clearly indicates a higher deamidation level of the egg proteins in mock-up G (31% for N, 57% for Q) compared to the egg proteins in J (5% for N, 1% for Q). Although factors such as the effect of different pigments upon the ageing behaviour of the protein binder remain unknown, it is interesting to note that the higher deamidation level occurs in the egg proteins in mock-up G, prepared approximately 80 years before mock-up J. Furthermore, the level of deamidation of egg proteins in J is very similar to the deamidation level observed in mock-ups A-D (5% for N, 1% for Q), prepared in 2005. The wool keratins in mock-up J show a much higher damage level (73% for N, 19% for Q), almost certainly due to the relatively harsh extraction conditions (high temperature, alkaline) used to extract the madder colorants from the dyed wool during the preparation of the lake. The influence of this treatment is also evident in the damage level of mock-up I (60% for N, 14% for Q), in which the only non-contaminant proteins detected were wool keratins. Taken together, these results highlight the importance of understanding the source of all the different protein components found in a sample, and illustrates how factors such as processing and ageing of the materials can influence the results obtained by proteomics.

Supplementary Table S2 - Percentage of deamidation of asparagine (N) and glutamine (Q) residues in the mock-ups and number of peptides on which the calculation is based. The values for samples for which the number of peptides used for the calculation is lower than 20 are reported in grey, to highlight that the calculation is not deemed confident in that case. For mock-ups A-H, all proteins were identified in the first MaxQuant search; for mock-up I, all proteins were identified in the second MaxQuant search; for mock-up J, the results of the two searches were merged (for details, see Section 1.1).

	**Sample**	**Peptides**		**Deamidation (%)**	
		**Small**	**Large**	**Small**	**Large**
**Asparagine (N)**	A	~~4~~	126	~~0.0~~	4.0
	B	103	239	5.8	4.6
	C	~~15~~	125	~~0.3~~	6.3
	D	173	228	7.0	7.3
	E	39	161	26.7	31.8
	F	~~0~~	~~16~~	-	~~78.3~~
	G	257	556	30.6	31.6
	H	~~6~~	~~3~~	~~0.0~~	~~32.4~~
	I	207	170	47.9	59.8
	J	1105	1142	12.0	9.9
**Glutamine (Q)**	A	~~2~~	150	~~0.0~~	0.1
	B	104	230	0.8	0.1
	C	24	108	1.0	0.4
	D	170	233	1.6	1.7
	E	48	174	1.1	0.8
	F	~~7~~	24	~~0.0~~	3.8
	G	250	527	57.4	55.8
	H	~~2~~	~~3~~	~~0.0~~	~~29.7~~
	I	195	149	24.7	14.0
	J	1078	1075	3.0	2.0

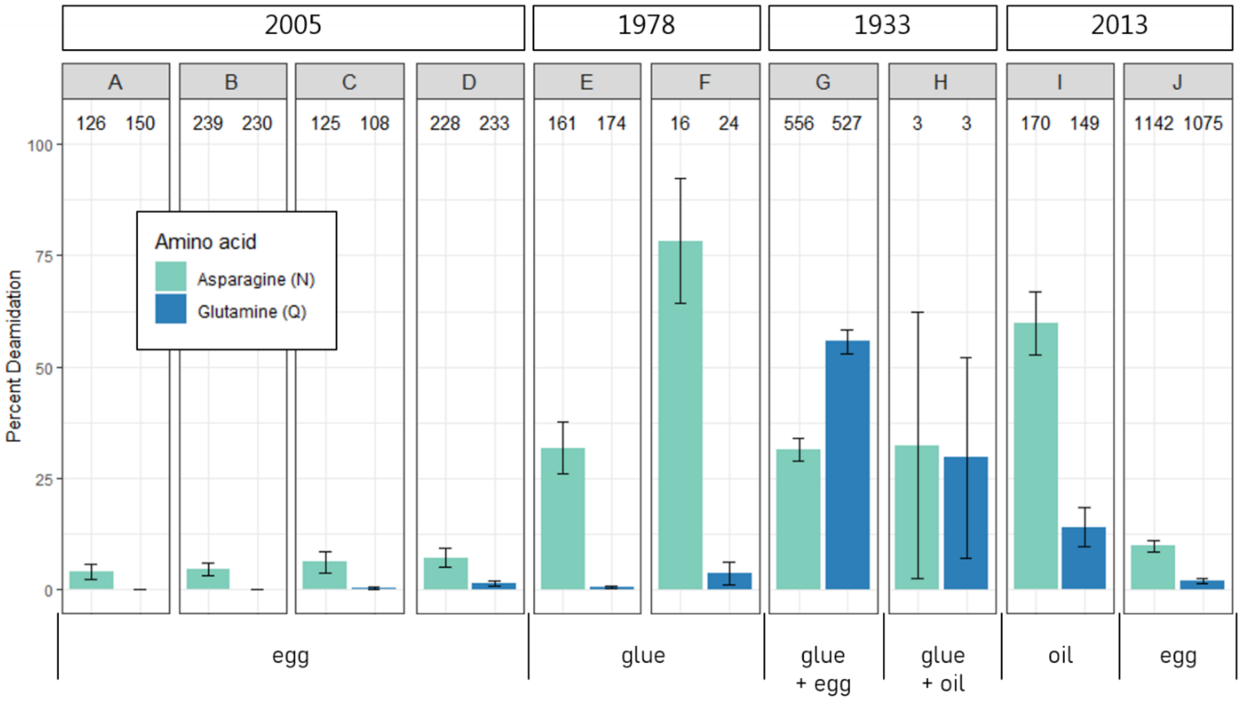

Supplementary Figure S2 - Percentage of deamidation of asparagine (N) and glutamine (Q) residues in the large samples of the mock-ups. Error bars indicate standard deviation around 1000 bootstrap replicates. Sample identifiers and the year of production of the paint are shown at the top, the number of peptides used for the calculation are indicated above each bar, and the paint binder is indicated at the bottom. For samples A-H, all proteins were identified in the first MaxQuant search; for mock-up I, all proteins were identified in the second MaxQuant search; for mock-up J, the results of the two searches were merged (for details, see Section 1.1).

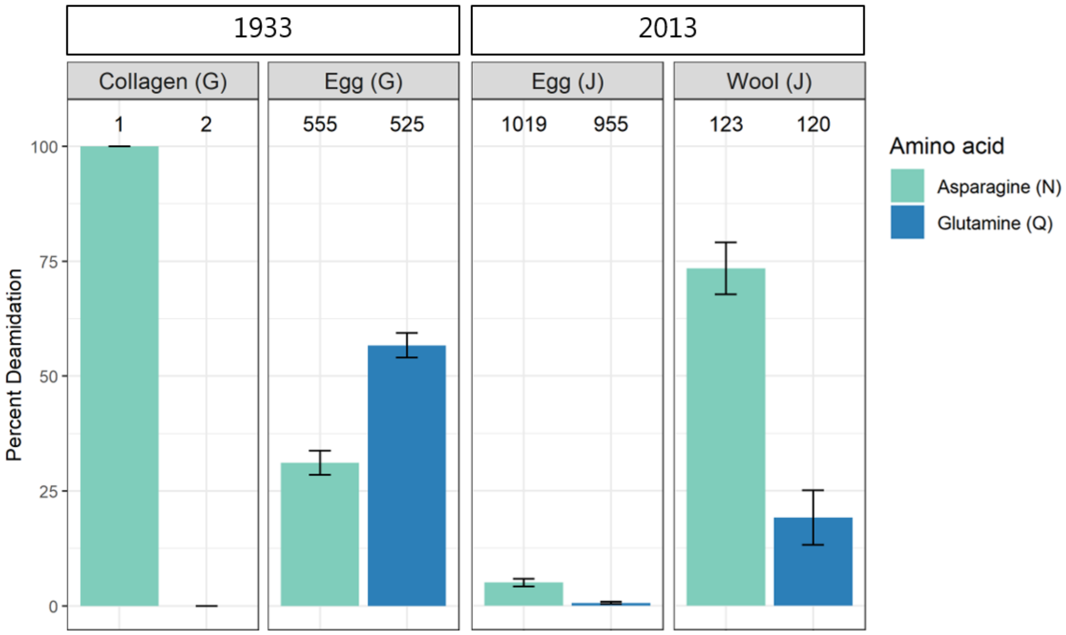

Supplementary Figure S3 - Percentage of deamidation of asparagine (N) and glutamine (Q) residues in collagen and egg proteins for the mock-up G (large sample), and for egg proteins and keratins for mock-up J (large sample). Error bars indicate standard deviation around 1000 bootstrap replicates. Sample identifier and protein class are shown in the grey boxes, the year of production of the paint is shown at the very top, while the number of peptides used for the calculation are indicated above each bar.

2. Summary of other analytical results (SEM-EDX and FTIR) for *The Virgin and Child with Saint John and an Angel*

Two samples (labelled IS2 and IS9) were removed from locations similar to those of 1:BP/1:GL and 2:YP, the blue cloak of the Virgin and the tallow drapery on the Angel's arm, respectively. The samples were embedded in polyester resin and prepared as cross-sections. Examination was carried out with an optical microscope in ordinary light and UV light (Supplementary Figure S4). In both cases, the stratigraphy was documented, showing a white ground layer, one or more paint layers, and a varnish layer, and the major inorganic components in the paint and preparation layers were confirmed by SEM-EDX analysis (Supplementary Table S3).

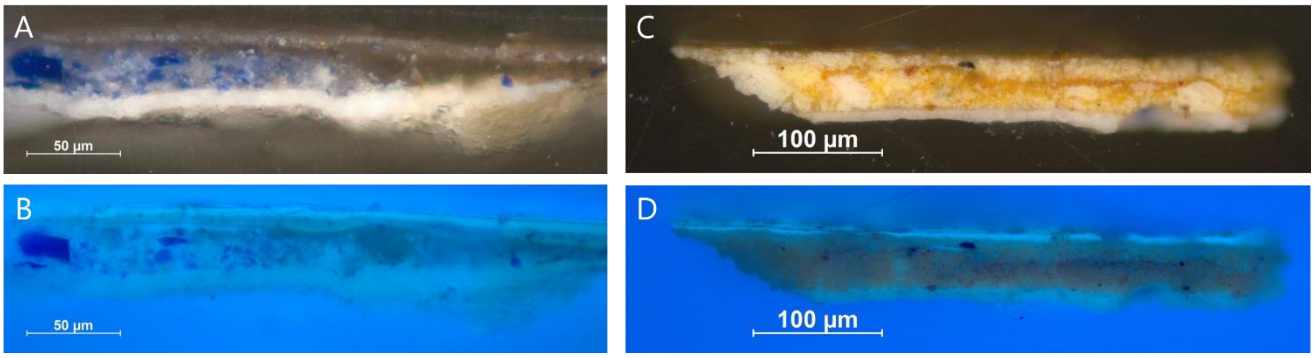

Supplementary Figure S4 - Cross-sections: sample IS2 at 50x magnification under visible light (A) and under UV illumination (B); sample IS9 at 20x magnification under visible light (C) and under UV illumination (D).

The three powdered samples taken for proteomic analysis (labelled 1:BP, 1:GL and 2:YP) were examined visually under the microscope and small, representative samples from each were compressed in a diamond cell and analysed by Fourier transform infrared (FTIR) microscopy (Supplementary Table S3). The results from the cross-sections and scrapings were then compared to ensure as much as possible was known about the inorganic materials in the samples before proteomics was performed.

Supplementary Table S3 - A summary of the SEM-EDX and FTIR results from samples obtained from The Virgin and Child with Saint John and an Angel, *attributed to the workshop of Sandro Botticelli. Where numbered, the layers are described from the surface layer downwards.*

**Samples analysed by SEM-EDX or FTIR**			
**Sample label**	**National Gallery sample name**	**Sample location**	**Inorganic materials**
**SEM-EDX analysis of cross-sections [elements detected (trace)]**			
--	IS2	Blue drapery (paint and ground layers)	1. Blue paint layer; ultramarine [EDX: Al, Si, Na, S, (K, Ca)] and lead white [EDX: Pb]<br>2. White imprimatura; lead white [EDX: Pb]<br>3. Ground; calcium sulphate [EDX: Ca, S, (Si)]
--	IS9	Yellow drapery (paint and ground layers)	1. Yellow/orange paint layers; predominantly lead-tin yellow [EDX: Pb, Sn] with some earths [EDX: Al, Si, Fe, K, Ca, Mg, P]<br>2. White underlayer; lead white [EDX: Pb]<br>3. Ground; calcium sulphate [EDX: Ca, S]
**FTIR analysis of scrapings Spatial information is lost, so location of materials inferred (main absorption bands)**			
**1:BP**	OS14	Blue drapery (paint with a little ground layer)	1. Surface crust; calcium carbonate (1402; 876 cm^{-1}) & oxalate (1323 cm^{-1})<br>2. Blue paint; ultramarine (1033 cm^{-1}) and lead white (1405; 840; 678 cm^{-1})<br>3. Ground; calcium sulphate dihydrate (3538; 3405; 1620; 1116; 670 cm^{-1})
**1:GL**	OS15	Blue drapery (ground layer)	1. Ground; Calcium sulphate dihydrate (3538; 3405; 1620; 1116; 670 cm^{-1})
**2:YP**	OS16	Yellow drapery (paint)	1. Surface crust; carbonate (1407 cm^{-1}; unidentified counterion) & calcium oxalate (1320 cm^{-1})<br>2. Earth pigments (1083; 1026; 910; 799 cm^{-1}). NB. lead tin yellow not infrared active

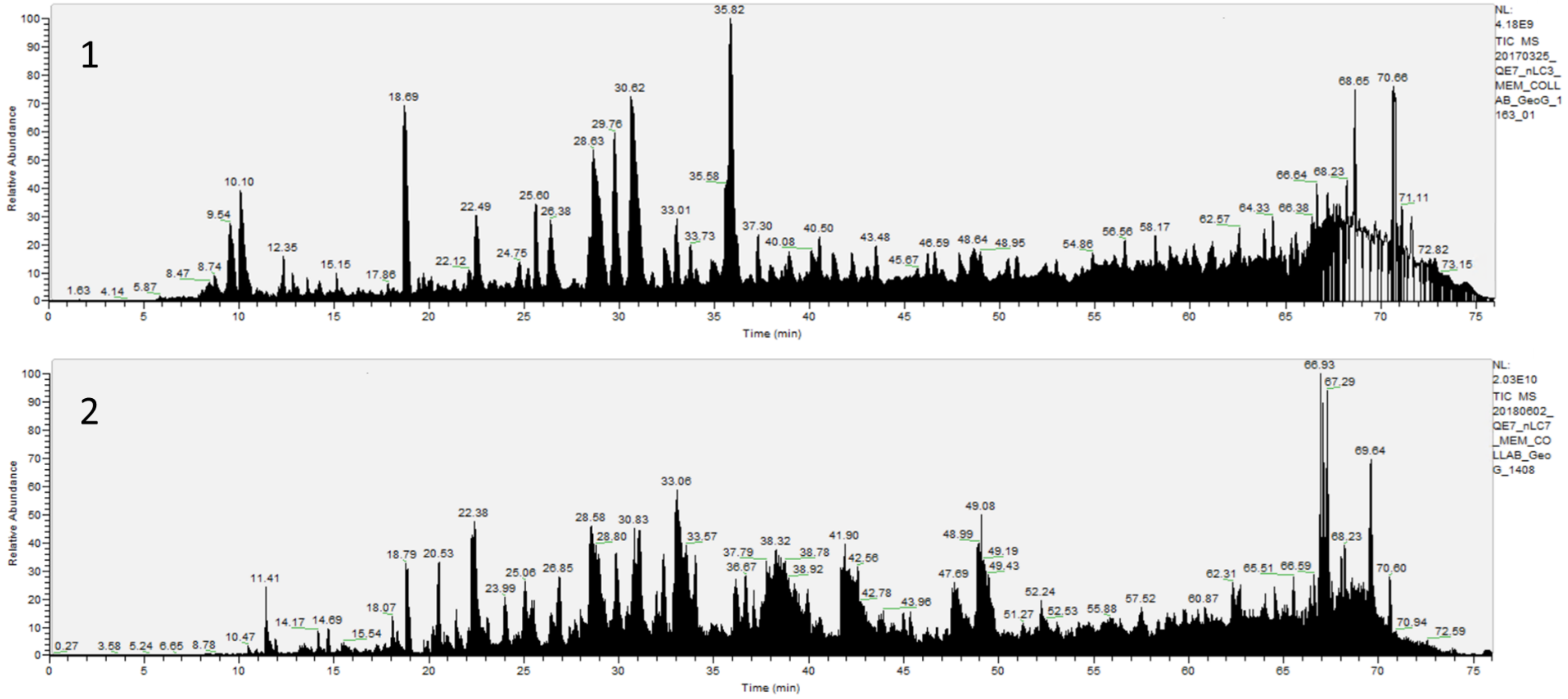

Supplementary Figure S5 - Total ion chromatograms of: 1) large sample from mock-up C; 2) sample 2:YP from The Virgin and Child with Saint John and an Angel.

Bibliography

1 Kirby, J., Spring, M. & Higgitt, C. The technology of red lake pigment manufacture: study of the dyestuff substrate. *National Gallery Technical Bulletin* **26**, 71-87 (2005).

2 Spring, M., Billinge, R. & Peggie, D. The technique and materials of two paintings from fifteenth-century Cologne in the National Gallery, London. *Zeitschrift für Kunsttechnologie und Konservierung: ZKK* **26**, p. 88-99 (2012).

3 Mazurek, J., Svoboda, M. & Schilling, M. GC/MS Characterization of Beeswax, Protein, Gum, Resin, and Oil in Romano-Egyptian Paintings. **2**, 1960-1985 (2019).

4 Consortium, U. UniProt: a hub for protein information. *Nucleic acids research* **43**, D204-D212 (2014).

5 Colombini, M. P., Andreotti, A., Bonaduce, I., Modugno, F. & Ribechini, E. Analytical strategies for characterizing organic paint media using gas chromatography/mass spectrometry. *Accounts of chemical research* **43**, 715-727 (2010).

6 Gautier, G. & Colombini, M. P. GC–MS identification of proteins in wall painting samples: a fast clean-up procedure to remove copper-based pigment interferences. *Talanta* **73**, 95-102 (2007).

7 Ren, F., Atlasevich, N., Baade, B., Loike, J. & Arslanoglu, J. Influence of pigments and protein aging on protein identification in historically representative casein-based paints using enzyme-linked immunosorbent assay. *Analytical and bioanalytical chemistry* **408**, 203-215 (2016).

8 De la Cruz-Canizares, J., Doménech-Carbó, M., Gimeno-Adelantado, J., Mateo-Castro, R. & Bosch-Reig, F. Suppression of pigment interference in the gas chromatographic analysis of proteinaceous binding media in paintings with EDTA. *Journal of Chromatography A* **1025**, 277-285 (2004).

9 Kenndler, E., Schmidt-Beiwl, K., Mairinger, F. & Pöhm, M. Identification of proteinaceous binding media of easel paintings by gas chromatography of the amino acid derivatives after catalytic hydrolysis by a protonated cation exchanger. *Fresenius' Journal of Analytical Chemistry* **342**, 135-141, doi:10.1007/BF00321708 (1992).

10 Cappellini, E. *et al.* Early Pleistocene enamel proteome sequences from Dmanisi resolve Stephanorhinus phylogeny. *bioRxiv*, 407692 (2018).

11 Robinson, N. E. & Robinson, A. *Molecular clocks: deamidation of asparaginyl and glutaminyl residues in peptides and proteins*. (Althouse press, 2004).

12 Ramsøe, A. *et al.* DeamiDATE 1.0: Site-specific deamidation as a tool to assess authenticity of members of ancient proteomes. *Journal of Archaeological Science* **115**, 105080 (2020).

13 Mackie, M. *et al.* Palaeoproteomic Profiling of Conservation Layers on a 14th Century Italian Wall Painting. *Angewandte Chemie International Edition* **57**, 7369-7374, doi:doi:10.1002/anie.201713020 (2018).

14 Cennini, C. *The Craftsman's Handbook (Il libro dell'arte)*. (Dover, 1933).
